# Doctors as Teachers: A Doctor-Led Education Programme to Improve Physical Health Escalation on Mental Health Wards

**DOI:** 10.1192/bjo.2026.11339

**Published:** 2026-06-30

**Authors:** Joseph Brougham, Mina Wahba, Siri Muraka, Shahid Qadar, Madeleine Upham

**Affiliations:** RDaSH, Doncaster, United Kingdom

## Abstract

**Aims:**

People with severe mental illness experience substantially higher premature mortality than the general population, much of which relates to physical health conditions. Mental health inpatient teams therefore need confidence and capability to recognise acute deterioration and escalate promptly. Evidence and local experience suggest mental healthnursing staff can feel under-prepared for acute physical health assessment and escalation, particularly for time-critical presentations such as chest pain (CP) and shortness of breath (SOB). This project was prompted by a real near-miss incident in which CP was not escalated appropriately to medical staff. We aimed to evaluate whether a focused, doctor-delivered teaching session improves nursing staff confidence in recognising and escalating CP and SOB in a mental health inpatient setting, and to support development of a wider ongoing physical health teaching programme.

**Methods:**

Medical staff delivered a brief interactive session addressing symptom recognition, red flags, initial assessment, and structured escalation. Participants completed pre- and post-session confidence ratings on a Likert Scale (with 1 meaning not at all confident, to 5 meaning very confident) in four domains: recognising CP, escalating CP, recognising SOB, and escalating SOB. Mean changes were analysed overall and by staff group (band 5 registered nurses, non-registered nursing staff, and nursing management).

**Results:**

Fifty-five staff participated (registered band 5 nurses n=20; non-registered nursing staff n=31; nursing management n=4). Confidence increased across all domains: recognising CP 3.25 to 4.36 (+1.11); escalating CP 3.15 to 4.27 (+1.13); recognising SOB 3.45 to 4.42 (+0.96); escalating SOB 3.15 to 4.29 (+1.15). The proportion reporting increased confidence was 76.4% (recognising CP), 74.5% (escalating CP), 69.1% (recognising SOB), and 72.7% (escalating SOB). Improvements were observed across all staff groups, with the largest gains among non-registered nursing staff, particularly for escalation.

**Conclusion:**

A short, doctor-led, ward-relevant teaching intervention was associated with meaningful improvements in mental health nursing staff confidence to recognise and escalateacute physical health presentations, addressing a key patient-safety risk highlighted by a near-miss. Following this intervention, doctor-delivered teaching was continued across additional physical health topics to build and maintain capability within the wider nursing team. The programme is now being rolled out to other services within the Trust, prompting a service evaluation to assess scalability and sustainability.

